# Predicting gene function using few positive examples and unlabeled ones

**DOI:** 10.1186/1471-2164-11-S2-S11

**Published:** 2010-11-02

**Authors:** Yiming Chen, Zhoujun Li, Xiaofeng Wang, Jiali Feng, Xiaohua Hu

**Affiliations:** 1 Computer School of National University of Defense Technology,Changsha,Hunan, China; 2 School of Computer Science and Engineering BeiHang University,BeiJing, China; 3 College of Information Science and Technology,Hunan Agricultural University,Changsha, China; 4 College of Information Engineering, Shanghai Maritime University, Shanghai, China; 5 College of Information Science and Technology, Drexel University, Philadelphia, PA, 19104, USA

## Abstract

**Background:**

A large amount of functional genomic data have provided enough knowledge in predicting gene function computationally, which uses known functional annotations and relationship between unknown genes and known ones to map unknown genes to GO functional terms. The prediction procedure is usually formulated as binary classification problem. Training binary classifier needs both positive examples and negative ones that have almost the same size. However, from various annotation database, we can only obtain few positive genes annotation for most offunctional terms, that is, there are only few positive examples for training classifier, which makes predicting directly gene function infeasible.

**Results:**

We propose a novel approach SPE_RNE to train classifier for each functional term. Firstly, positive examples set is enlarged by creating synthetic positive examples. Secondly, representative negative examples are selected by training SVM(support vector machine) iteratively to move classification hyperplane to a appropriate place. Lastly, an optimal SVM classifier are trained by using grid search technique. On combined kernel ofYeast protein sequence, microarray expression, protein-protein interaction and GO functional annotation data, we compare SPE_RNE with other three typical methods in three classical performance measures recall *R,* precise *P* and their combination *F*: twoclass considers all unlabeled genes as negative examples, twoclassbal selects randomly same number negative examples from unlabeled gene, PSoL selects a negative examples set that are far from positive examples and far from each other.

**Conclusions:**

In test data and unknown genes data, we compute average and variant of measure *F*. The experiments showthat our approach has better generalized performance and practical prediction capacity. In addition, our method can also be used for other organisms such as human.

## Background

One of the important challenges in the post-genome era is determining the functional role of all genes in the cell although about one-third of the genes have been annotated and deposited in database such GO(gene ontology) [1]. With the recent invention of several large-scale experimental methods, a wealth of functional genomic data was accumulated, including sequence, micro-array expression profile and protein-protein interaction data. These large data-sets have fueled an interest in computational approaches to gene function prediction, which promises to harness the information present in these large collections of data to automatically deduce accurate gene annotations [2,3]. Furthermore, many works have shown that integration of different kinds of data sources can considerably improve prediction results [4,5]. GO is a widely-used set of functional terms with which some genes are annotated, we also call functional terms as functional classes in related to classification problem from machine learning. GO functional annotation associates each gene or gene product to some functional terms. For an unknown gene, predicting its functions will assign some GO functional terms to it, which is called multi-label classification problem in machine learning community. The mainstream approach is to transform it into a binary classification task for each functional class, which focuses on training a classifier such as SVM (support vector machine) with some labeled positive and negative examples. However, the available information from the annotation databases, such as GO [1], is only about positive examples, i.e. for a functional class, we only know which gene is assigned to it, but we are not sure that a gene has no this function except for too few genes. As a result, when training classifier for a functional class, we can only obtain labeled positive examples and many unlabeled ones. In other words, for a functional class, we need to learn a classifier from positive and unlabeled examples. Thus, an important step is to select a suitable set of negative examples from unlabeled examples before training classifier.

Some approaches to select negative examples have been proposed. For example, Lanckriet et. al labeled the annotated genes as positive examples and the remaining ones as negative ones for each functional class [4]. Carter et.al randomly selected the negative examples with the same size as the positive examples from the unlabeled examples [6]. We call these two methods *twoclass* and *twoclassbal* algorithm respectively. Chunlin Wang et.al selected a set of negative examples in two steps: firstly, identifying genes which are far from each other and the most dissimilar to positive examples as initial negative examples set. Then, using iteratively SVM to expanse negative examples and stopping while the remaining unlabeled examples are less than given threshold. Their method is called PSoL(Positive Sample only learning) and its detail can be found in [7].

Above approaches can be divided into two categories and some problems can occur when only few positive examples and major unlabeled ones are given: 1. Regarding all unlabeled examples as negative examples [4]. On the one hand, it may lead to class imbalance problem because of few positive examples [8]. on the other hand, the false negative noise may seriously decrease the prediction accuracy. 2. Selecting negative examples with same size as positive examples [6,7]. These methods eliminate the impact of imbalanced problem, but, only few negative examples can be selected, as a result, the classifier is trained on a small training set and easily leads to over-fitting. When we use GO annotation, many functional classes have few annotated genes, which will lead to a lower prediction accuracy and need to be solved [9]. In this paper, aiming at both imbalance and over-fitting problem for genes function prediction with only few positive examples and unlabeled examples, we propose a novel strategy for predicting genes function using SVM. Firstly, we create some synthetic positive examples with few negative noises to enlarge positive examples set *P*. Secondly, we extract a representative negative example set *RN* from unlabeled genes *U* using SVM iteratively. Finally, an optimal SVM classifier with RBF (Radial Basis Function) is trained by using Grid-search technique. This method is called SPE_RNE(Learning classifier by Synthetic Positive Examples and Representative Negative Examples).

## Results and discussion

### Experiment setting

#### Data sets

##### Gene annotation

We used gene ontology and corresponding gene function association of Yeast [10] released in April 2007. Gene association file contained 5,873 genes ,the number of known and unknown genes is 3,796 and 2,077 respectively. We up-propagate the gene annotation along GO hierarchical structure and obtained a reduced GO which has only 99 GO terms under guidance of biological experts. To compare the algorithm performance, we divide them into four groups according to number of annotated genes as shown in Table 1. There are 53 functional classes with annotated genes less than 60 among total 99 terms.

**Table 1:** Four groups of function terms and number of term

*annotation interval*	< 60	60-100	100-300	> 300
*number of terms*	53	17	18	11

99 functional terms are divided into four groups according to number of annotated genes. The first row are four groups with interval of genes number. The second row is number of terms.

##### Protein sequence

The protein sequence of all of the Yeast genes were downloaded from SGD [10].We applied the Smith-Waterman pairwise sequence alignment algorithm [11] to these sequences. Each protein is represented as a vector of Smith-Waterman log E-values, and computed with respect to all 5,873 Yeast genes. A 5873*5873 similar matrix is obtained.

##### Microarray expression profile

Microarray datasets are real-valued matrices measuring gene expression levels under different experimental conditions. We use gene expression microarray data from the Stanford Microarray Database(SMD) [12] containing results from several publications, providing a total of 294 real-valued features for all 5,873 genes. Microarray entries typically include missing values due to experimental imperfections. We estimate such entries using the widely accepted KNNimpute algorithm [13] with default *k* value. Then, we computed similarity between two genes using Gauss kernel with *γ* = 2. The second 5873*5873 gene similar matrix is generated.

##### Protein–protein interaction

We downloaded the protein-protein interaction data from BioGRID2.0.30 [14]. Protein-protein interaction data is described as a graph in which nodes denote protein and edges denote interaction and diffusion kernel [15] with diffusion constant *β* = 2 is used to measure the similarity between two proteins. Each gene is also represented as a vector of similarity with respect to all 5,873 genes. The third gene similar matrix is computed.

Several previous researches have shown that integrating various genomic data to predict gene function can improve prediction accuracy [4,5]. In this paper, we add three similar matrices and obtain a sum matrix. It is noticeable that each matrix should be centralized and normalized to eliminate the effect from major data before adding them [4]. While training SVM classifier, this pre-computed kernel matrix is used.

#### Experiment setting and evaluation

We used LIBSVM [16] to implement SPE-RNE and related algorithms two class SVM twoclass, two class balanced SVM (twoclassbal)and PSoL in matlab. First, we divided 3796 known genes into training set and validation set, after training SVM classifier on training set, the generalized performance of algorithms were compared on validation set. Widely-accepted measures, including precision rate *P*, recall rate *R* and their combination *F*1, are used. Their definitions are as follows:

            (1)

              (2)

               (3)

where *TP,FP* and *FN* denote the number of true positive, false positive and false negative respectively. Then, using 2,077 unknown genes released in April 2007 as test examples, we predict their functions and evaluate ROC (Receiver operating characteristics) score with gene function association released in December 2008 as annotation standard.

### Performance comparison on known genes

For each functional class, 3796 genes are in two categories: genes assigned to this functional class and unlabeled genes. we randomly select 20 percent from these two categories as validation set, the others are training set. SVM classifiers are learned on training set and used to predict genes functions on validation set to evaluate generalized performance of algorithms.

When the number of negative examples is far more than positive examples, imbalanced problem occurs and the algorithm can not recall any true positive examples for some functional classes. As a result, *P* = 0 and *R* = 0 result in *F*1 = *NaN* (Not a Number) in matlab. Table 2 shows the number of *NaN* for each functional group.

**Table 2:** The number of functional class whose *F*1 = *NaN* for four algorithms and four functional groups

*algorithms*	< 60	60-100	100-300	> 300
twoclass	31	8	3	1
twoclassbal	0	0	0	0
PSoL	22	6	0	1
SPE-RNE	0	0	0	0

When the number of negative examples is far more than positive examples, imbalanced problem occurs and the algorithm can not recall any true positive examples for some functional classes. As a result, *P* = 0 and *R* = 0 result in *F*1 = *NaN* (Not a Number) in matlab. Table 2 shows the number of *NaN* for each functional group.

As shown by table 2, twoclass has the most serious imbalance and PSoL has more serious imbalance, but our method SPE-RNE, like twoclassbal, doesn’t suffer from imbalanced problem at all because we select reasonable quantity of negative examples after enlarging the positive examples set. In addition, functional classes with few annotated genes have more serious imbalance.

For twoclassbal algorithm, while serious imbalance does not occur, the over-fitting may arise to affect prediction performance due to few training examples. To evaluate the algorithm fairly, we set *F*1 = *NaN* to *F*1 = 0. For each algorithm and functional group, the means and variances of *F*1 are listed in Table 3 and Table 4 respectively.

**Table 3:** The mean of *F*1 for four algorithms and four functional groups

*algorithms*	< 60	60-100	100-300	> 300
twoclass	0.1679	0.1881	0.3641	0.3675
twoclassbal	0.0912	0.1329	0.2877	0.3785
PSoL	0.2094	0.2223	0.4006	0.3999
SPE-RNE	0.5545	0.6589	0.5639	0.4848

Table 3 lists the average values of *F*1 for all algorithms on four functional terms groups. These values can evaluate the comprehensive prediction performance of algorithms on different groups and compare their generalized capacity.

In table 3, although twoclassbal has not class imbalance problem, but it has worst performance because it has too few training examples, which easily causes over-fitting. For functional classes with few annotated genes, the existed algorithms, like twoclass, twoclassbal, PSoL have lower *F*1 values, our algorithm significantly improves the *F*1 values in this case. For functional classes with more annotated genes, our algorithm has better performance too. While only few training examples are used to learn SVM classifier, over-fitting problem may occur and make algorithms unstable. We compute the variances of *F*1 for each functional group to evaluate the stability of algorithm. Table 4 shows that our algorithm has good stability.

**Table 4:** The variance of *F*1 for four algorithms and four functional groups

*algorithms*	< 60	60-100	100-300	> 300
twoclass	0.0636	0.0929	0.1034	0.0536
twoclassbal	0.0142	0.0083	0.0462	0.0215
PSoL	0.0594	0.0813	0.0629	0.0456
SPE-RNE	0.0523	0.0485	0.0359	0.0420

Table 4 lists the variances of *F*1 for all algorithms on four functional terms groups. These values can evaluate the stability of algorithms on different groups. While only few training examples are used to learn classifier, over-fitting may occur and makes algorithm unstable.

### Predicting performance on unknown genes

Since April 2007, some of 2077 unknown genes have been annotated with some functions. We consider these 2077 genes as test examples and use trained SVM classifier to predict function for them. The GO function association released in December 2008 is regarded as complete annotation, that is, for each functional class, if a gene is assigned to it, the label is set to 1, otherwise -1. For each algorithm, the ROC score, which is area under ROC, is evaluated as comparison measures. In previous section, we use *F*1 to evaluate algorithm performance because we think that GO function association released in April 2007 is incomplete. The ROC scores are listed in Table 5.

**Table 5:** The average ROC score for four algorithms and four functional groups

*algorithms*	< 60	60-100	100-300	> 300
twoclass	0.5081	0.7448	0.7743	0.7044
twoclassbal	0.5207	0.7529	0.8002	0.7118
PSoL	0.5313	0.7626	0.7726	0.7142
SPE-RNE	0.6827	0.7969	0.8084	0.7266

ROC score, that is area under ROC curve, is a widely-accepted performance measure for prediction classification problem. The larger ROC score is, the better the performance of classification algorithm is.

**Fig. 1:**
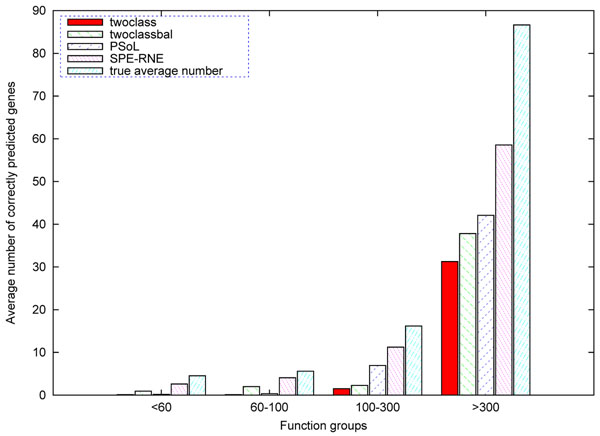
**The average number of correctly predicted genes according to GO association released in December 2008 for unknown gene in April 2007** In figure 1, the height of bar denotes the average number of genes predicted correctly by four algorithms and average true number of genes on different groups.

For group 1 and 2, our algorithm significantly improve the ROC score, which illustrates better prediction performance for unknown genes. For group 3, we only add synthetic examples as many as positive examples, and for group 4, we don’t create any synthetic positive examples. But, our algorithm for extracting representative negative examples slightly improve the ROC score too. The average number of correctly predicted genes and true average number are displayed in Figure 1 for each group. In each group, our algorithm can recall more positive genes on average.

### Predicting result on unknown genes

We list predicted functional classes for ten genes with most predicted functional terms in Table 6, these genes were unknown in April 2007, but they were annotated with one or multiple functional classes in December 2008.

**Table 6:** The ten genes with the most predicted functions and their predicted functions

*protein*	*predicted functions*			
YKR084c	GO:0000166	GO:0003676	GO:0003824	GO:0003924
	GO:0005488	GO:0016462	GO:0016787	GO:0017076
	GO:0017111	GO:0045182		
YEL030w	GO:0000166	GO:0003824	GO:0005488	GO:0005515
	GO:0016787	GO:0017076	GO:0017111	GO:0051082
YDR332w	GO:0003676	GO:0003824	GO:0004386	GO:0016462
	GO:0016787	GO:0016818	GO:0016887	GO:0017111
YBR025c	GO:0000166	GO:0003824	GO:0016462	GO:0016818
	GO:0016887	GO:0017076	GO:0017111	
YMR078c	GO:0003676	GO:0003824	GO:0016787	GO:0016818
	GO:0016887	GO:0017111		
YLR035c	GO:0000166	GO:0003676	GO:0003677	GO:0003824
	GO:0016787	GO:0017076		
YGL175c	GO:0003676	GO:0003677	GO:0003824	GO:0004519
	GO:0016787	GO:0016788		
YLR419w	GO:0003676	GO:0003824	GO:0004386	GO:0016818
	GO:0016887	GO:0017111		
YNL001w	GO:0003824	GO:0004518	GO:0004519	GO:0004540
	GO:0016788			
YJL057c	GO:0003824	GO:0004672	GO:0016301	GO:0016740
	GO:0016772			

Table 6 lists ten genes with top predicted functional classes and their predicted functional terms. The first column is gene name and other columns are functional classes predicted correctly by algorithm SPE-RNE.

## Conclusions

In this paper, We propose a novel approach to predicting gene function for genes with few positive examples and unlabeled ones SPE-RNE: creating synthetic examples to enlarge the set of positive examples, extracting representative negative examples from unlabeled examples and training SVM classifier using Grid-search technique. For SPE-RNE, the validation on known gene data set shows its best *F*1 value and good stability. Prediction on unknown genes set illustrates its higher ROC scores and better prediction performance than several classic algorithms. All the algorithms run in a sum matrix which is obtained by adding simply several similarity matrixes from heterogeneous data sources, which may loss some information. How to integrate effectively these heterogeneous data to predict gene function is our next research subject in future. In addition, our method can also be used for other organisms such as human.

## Methods

### Creating synthetic examples to enlarge the positive examples set

The problem about learning classifier with few positive examples can be found in text classification domain. An intuitive idea is to enlarge the positive examples set. Li et al [17] assume that positive examples in *P* and likely positive examples from *U* have common underlying feature dimensions (or subspaces) as they belong to the same class. The representative words (*RW*) extracted from *P* are used to identify more hidden positive examples from *U*. Fung et al [18] firstly identified feature words in *P* and select a set of reliable negative examples from *U*, then, all negative examples are divided into some clusters due to the diversity of negative examples, for an example from *U*, computing similarity *d_P_* between it and centroid of positive examples and similarity *d_U_* between it and cluster centroid of negative examples, if *d_U_ – d_P_* is greater than a given threshold, it is added to *P*.

Due to sparse and discrete features of text vectors, Li and Fung’s method can not be used for continuous feature vector of gene. We can not find so-called feature words in gene vectors to identify hidden positive examples. We can only use some distance, such as Euclidean distance, to measure the similarity between genes [7]. Fung’s method enlarges the positive examples set after selecting the reliable negative examples, but, if we use Euclidean distance or cosine distance, a better positive example centroid can not be found due to irregularity of positive examples distribution, which has been validated by our many experiments. Our experiments also show that enlarging the *P* by identifying hidden positive examples from *U* generates easily false positive noise because of few hidden positive examples in *U.*

We create synthetic positive examples to enlarge the *P*. For each **e** ∈ *P*, we find its *k* nearest neighbors in *P* and then create synthetic examples along the line segments joining e and some/all of the *k* nearest neighbors. Depending on the required amount of enlarging, *n* neighbors from the *k* nearest neighbors are randomly chosen. For instance, if the needed amount of enlarging is 500%, only five neighbors from the *k* nearest neighbors are chosen and one synthetic example is generated in each direction. Given **e**′ ∈ *P* is one of *k*-nearest neighbors of **e**, *α* ∈ (0, 1) is a random number, Synthetic examples are generated in the following way:

 (4)

The following propositions convinces us of likely positive examples of .

#### Proposition 0.1

*Let***e***and***e**′ *be two positive examples. Then,*

is a likely positive example.

#### Proof

Let the classification hyperplane be

*f*(**x**) = **w***^T^***x** + *b*

According to Vapnik’s theory minimizing empiric risk [19], following two inequalities are correct with probability close to 1:

**w***^T^***e** + *b* > 0

**w***^T^***e**′ + *b* > 0

*Further f*(*α***e** + (1 – *α*)**e**′)

= **w***^T^*(*α***e** + (1 – *α*)**e**′) + *b*

= *α***w***^T^***e** + (1 – *α*)**w***^T^***e**′ + *b*

= *α*(**w***^T^***e** + *b*) + (1 – *α*)(**w***^T^***e**′ + *b*)

Thus

*f* (*α***e** +(1 – *α*)**e**′) > 0

That is,  = *α***e** + (1 – *α*)**e**′ is a positive example with probability close to 1.

Therefore, the synthetic example  is probably positive example and the enlarged set of positive examples,  has few negative noise. In fact, from the point of view of algebra,  is convex combination of **e** and **e**′, and from the point of view of geometry,  is a random point in line segment from **e** to **e**′. We have following algorithm 1 for creating synthetic examples.

In our experiments, all the functional classes are divided into four groups according to number of annotated genes. We enlarge positive example set with different times for different groups and set *k* = 10, which is shown in Table 7. Particularly, we did not make synthetic positive examples for functional classes with more than 300 annotated genes.

**Table 7:** The enlarging times for different functional groups

*annotation interval*	< 60	60-100	100-300	> 300
*enlarging times*	4	2	1	0

Algorithm SPE-RNE enlarges the positive examples set with different times according to the number of positive examples. The first row is functional groups and the second row is times. The functional classes with few positive examples enlarges more times.

#### Algorithm 1

Algorithm creating synthetic examples

1: **function** MAKESYNEXPS(*P,n,k*)

2: *P is positive examples set*;

3: *n is amount of enlarging*

4: *k is number of nearest neighbors*

5: pnum=size of *P*

6: nnarray=array of nearest neighbor with size *k*

7: synexps=array of synthetic examples

8: *making syntheticpositive examples*

9: **for all e** ∈ *P***do**

10: nnarray=*k* nearest neighbors of **e** from *P*

11: selnn=*n* nearest neighbors randomly selected from nnarray

12: **for all e**′ ∈ selnn **do**

13: generating a random number *α* ∈ (0, 1)

14:  = *α* * **e** + (1 – *α*) * **e**′

15: adding **e** into synexps

16: **end for**

17: **end for**

18: Return synexps;

19: **end function**

### Extracting the representative negative examples

After enlarging the positive examples set *P*, we need to train SVM on new positive examples set  and unlabeled examples set *U* . To learn a better classifier, we should extract a subset of the most probably negative examples from unlabeled data *U* so that it can best recover the positive examples hidden in *U* . This extracted negative examples subset can represent the whole negative set well and should have suitable size to avoid the class imbalance problem. To achieve this goal, our algorithm extracts representative negative examples and consists of three steps.

Step 1, identifying a reliable initial negative examples set. For gene vectors, only distance-based similarity can be used and the most dissimilar genes to positive examples are assumed as reliable negative examples. In PSoL, initial negative set *N* met two conditions:  all elements in *N* are most dissimilar to positive example set .  elements in *N* are far from each other. Since this problem is NP-hard, an approximate solution was used. In our algorithm, one-class SVM [20] is utilized to extract efficiently initial negative examples. Give a percentage of negative examples, such as 10 percent, it can draw an initial decision boundary to cover most of the positive and unlabeled examples. The data points not covered by the decision boundary can be regarded as negative example points because these data points are far from the positive set in the feature space.

Step 2, learning SVM iteratively to move classification hyperplane to an appropriate place. In this iteration process, a set of classifier, *C_S_* and corresponding set of negative example set, *N_S_* are obtained. In *i^th^* iteration, we obtain not only SVM classifier *C_i_* but also negative example set *N_i_*_+1_ to be used to train next SVM

*N_i_*_+1_ = *N_pred_* ∪ *N_SVs_*  (5)

where *N_pred_* is the most reliable negative examples predicted in current iteration and *N_SVs_* is negative support vector set of *C_i_*. To avoid class imbalance, the size of *N_pred_* is set as *N_pred_* ≤ *m*|| and many experiments shown that *m* = 3 is suitable. In addition, only the negative support vectors of *C_i_* are selected as representatives of previous negative training examples. In this iteration process, *|U*| becomes smaller and smaller. When only few unlabeled examples are remained, the *N_i_* may has more false positive examples and the classifier may become bad, therefore, we stop iterating. According to a large number of experiments, the stopping criteria is set as:

 (6)

**Fig. 2:**
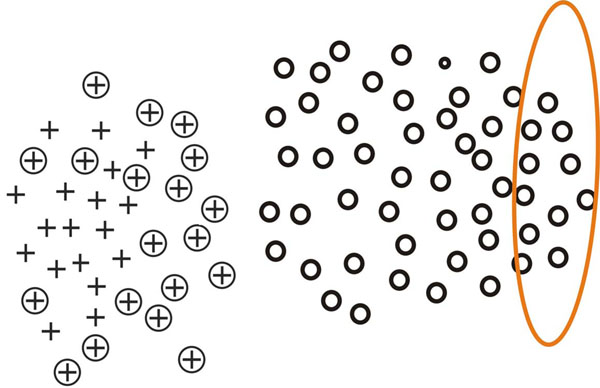
**One-class SVM extracts initial negative example set** In figure 2, plus signs, plus signs with circle and circles denote positive examples, potential positive examples and unlabeled examples respectively. The points covered by ellipse are negative examples set *N*_0_ and the line is classification hyperplane. One-class SVM is utilized to extract the initial negative examples. Give a percentage of negative examples, such as 10 percent, it can draw an initial decision boundary to cover most of the positive and unlabeled data. The data points not covered by the decision boundary can be regarded as negative data points because these data points are far from the major positive set.

**Fig. 3:**
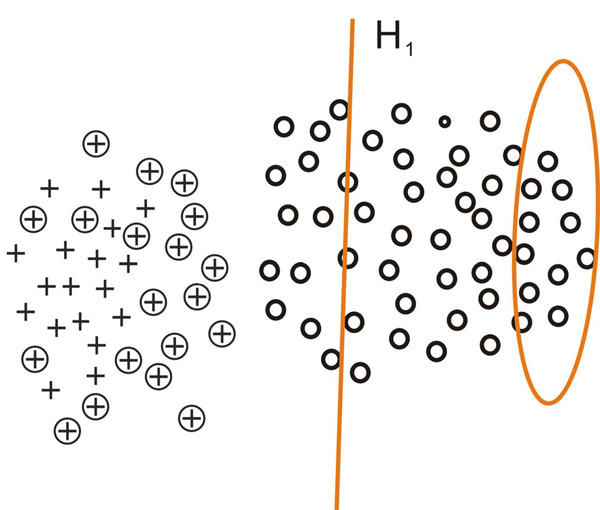
**The first iteration in which the negative example set *N*_1_ is obtained by moving the classification hyperplane towards positive example set** In figure 3, plus signs, plus signs with circle and circles denote positive examples, potential positive examples and unlabeled examples respectively. The points covered by ellipse are negative examples set *N*_1_ and the line is classification hyperplane. With the positive examples  and initial negative example set *N*_0_,the SVM classifier *C*_0_ is learned, the negative example set *N*_1_ consists of the support vectors of *C*_0_ and the unlabeled examples predicted as negative examples.

**Fig. 4:**
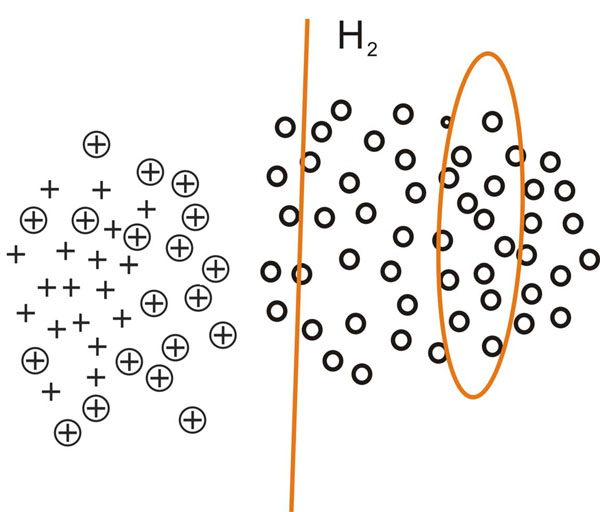
**The negative example set *N*_2_ is obtained with the second iteration** In figure 4, plus signs, plus signs with circle and circles denote positive examples, potential positive examples and unlabeled examples respectively. The points covered by ellipse are negative example set *N*_2_ and the line is classification hyperplane.With the new training set  ∪ *N*_1_, the SVM classifier *C*_1_ is learned, the negative example set *N*_2_ consists of the support vectors of *C*_1_ and the unlabeled examples predicted as negative examples.

**Fig. 5:**
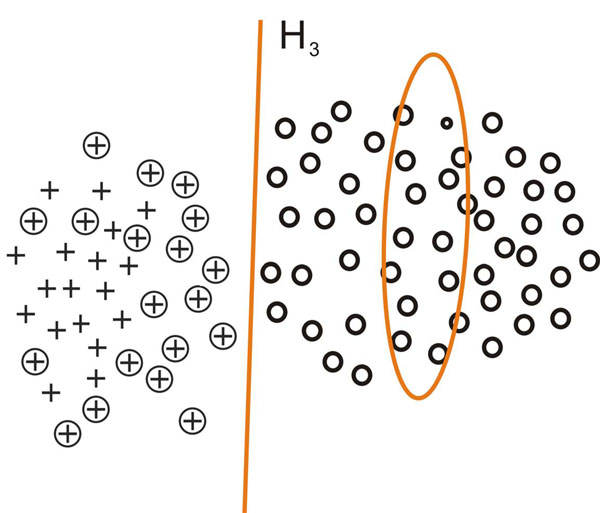
**Obtaining the negative example set *N*_3_ with the more iteration** In figure 5, plus signs, plus signs with circle and circles denote positive examples, potential positive examples and unlabeled examples respectively. The points covered by ellipse are negative example set *N*_3_ and the line is classification hyperplane.With the new negative example set *N*_2_, the SVM classifier *C*_2_ is learned, the negative example set *N*_3_ consists of the support vectors of *C*_2_> and the unlabeled examples predicted as negative examples. The process can proceed until the condition *|U*| ≤ 4 * || is meet.

This process is inspired by [21] and can be intuitively demonstrated using figure 2, 3, 4, 5. In figure 2, 3, 4, 5, plus signs, plus signs with circle and circles denote positive examples, potential positive examples and unlabeled examples respectively. The points covered by ellipse are negative examples set *N_i_* and the line is classification hyperplane. Figure 2 demonstrates how one-class SVM extracts initial negative examples. Figure 3, 4, 5 illustrate three iterations in which the classification hyperplane moves towards positive examples set.

Step 3, selecting the best representative negative set. The representative negative set should have best classification performance, therefore, we use each SVM from *Cs* to classify a validation set *V* that is selected randomly form  and *U* with 10 percent of total respectively at the start of algorithm. The discrimination ability of the trained classifiers is evaluated with *F*1 . Accordingly, the negative set corresponding to the best classifier is returned as the representative negative samples *RN*. In stead PSoL selects final classifier to classify remaining examples, our algorithm selects best classifier according to classification performance on validation set and corresponding negative set is regarded as representative negative set, final classifier is trained in the third stage. The algorithm for extracting representative negative examples is displayed in Algorithm 2.

#### Algorithm 2

Algorithm selecting representative negative examples

1: **function** SELNEGEXPS(, *U*)

2: randomly selecting 10 percent of  and *U* respectively as validation set *V*

3:  = *– V*, *U*′ = *U – V*;

4: *identifying the initial reliable negative examples*

5: Training one-class SVM classifier *C*_0_ based on  and *U′*;

6: Classify *U*′ using *C*_0_. The predicted negative set *N*_1_ is used as the initial negative training set

7: *U*′ = *U*′ *– N*_1_

8: *Training iteratively SVMs.*

9: Classifier set *Cs*;

10: negative set *Ns*

11: *i* = 1

12: **while** |*U*′| ≥ 4 *|| **do**

13: Training two-class SVM classifier *C_i_* based on  and *N*_1_;

14: *Cs(i)* = *C_i_, Ns*(*i*) = *N*_1_;

15: Classify *U*′ by *C_i_*, *N*_2_ is the predicted reliable negative set, where |*N*_2_| ≤ *m*||;

16: *N*_1_ = *N*_2_ + *N_SV_*, where *N_SV_* is the negative SVs of *C_i_*;

17: *U′* = *U′* – *N*_2_.

18: *i* = *i* + 1;

19: **end while**

20: *selecting representive negative examples set*

21: **for all***C* ∈ *Cs***do**

22: computing the *F*1 on *V*

23: **end for**

24: return *RN* from *Ns* with maximum *F*1

25: **end function**

### Training the SVM for predicting genes function

After enlarging the positive examples set and extracting the representative negative examples, we merge these two kinds of examples into a training set  ∪ *RN*. A SVM classifier with RBF kernel is trained on it. Grid-search technique [22] is used to search the optimal parameters *c* and *g* and an optimal SVM classifier can be successfully obtained.

## Competing interests

The authors declare that they have no competing interests.

## Authors' contributions

Zhoujun Li, Xiaofeng Wang and Jiali Feng conceived this research and applied project. Xiaohua Hu put forward some suggestion about two algorithms and revised the manuscript. Yiming Chen designed and coded algorithms, wrote the manuscript. All authors read and approved the final manuscript.
